# A novel de novo *KDM5C* variant in a female with global developmental delay and ataxia: a case report

**DOI:** 10.1186/s12883-021-02380-9

**Published:** 2021-09-16

**Authors:** Natalie C. Lippa, Subit Barua, Vimla Aggarwal, Elaine Pereira, Jennifer M. Bain

**Affiliations:** 1 Institute for Genomic Medicine, Columbia University Irving Medical Center, New York, NY USA; 2 Department of Pathology and Cell Biology, Columbia University Irving Medical Center, New York, NY USA; 3 Department of Pediatrics, Columbia University Irving Medical Center, New York, NY USA; 4 Department of Neurology, Columbia University Irving Medical Center, New York, NY USA

**Keywords:** Ataxia, Neurodevelopmental disorder, *KDM5C*, Intellectual disability, Case report

## Abstract

**Background:**

Pathogenic variants in *KDM5C* are a cause of X-linked intellectual disability in males. Other features in males include short stature, dysmorphic features, seizures and spasticity. In some instances, female relatives were noted to have learning difficulties and mild intellectual disabilities, but full phenotypic descriptions were often incomplete. Recently, detailed phenotypic features of five affected females with de novo variants were described. (Clin Genet 98:43–55, 2020) Four individuals had a protein truncating variant and 1 individual had a missense variant. All five individuals had developmental delay/intellectual disability and three neurological features.

**Case presentation:**

Here we report a three-year-old female with global developmental delay, hypotonia and ataxia. Through whole exome sequencing, a de novo c.1516A > G (p.Met506Val) variant in *KDM5C* was identified. This missense variant is in the jumonji-C domain of this multi domain protein where other missense variants have been previously reported in KDM5C related disorder. The KDM5C gene is highly intolerant to functional variation which suggests its pathogenicity. The probands motor delays and language impairment is consistent with other reported female patients with de novo variants in *KDM5C*. However, other features reported in females (distinctive facial features, skeletal abnormalities, short stature and endocrine features) were absent. To the best of our knowledge, our proband is the first female patient reported with a diagnosis of ataxia.

**Conclusions:**

This case report provides evidence for an emerging and phenotypic variability that adds to the literature of the role of *KDM5C* in females with neurodevelopmental disorders as well as movement disorders.

## Background

The lysine specific demethylase 5C (*KDM5C*) is a cause of X-linked intellectual disability (ID) in males (Mental retardation, X-linked, syndromic, Claes-Jensen type; MIM# 300534). The gene was first described to cause disease in 2005 by Jensen et al. who identified seven *KDM5C* variants in 20 affected males from 7 families through a mutational screen of brain expressed genes on the X chromosome in a cohort of families with X-linked ID [1]. Following this initial publication, additional reports were published linking this gene to disease [2, 3]. These reports indicated that some proband’s mothers and other female relatives who carried the *KDM5C* variant could also be affected, but the phenotypic descriptions were lacking.

The first de novo variant reported in a female was identified in a patient with spastic diplegia, speech dyspraxia and ID through trio exome sequencing [4]. The second case was a presumed de novo (dad unaffected but unavailable for testing) 0.4 Mb deletion encompassing six genes in a female with severe ID, no speech and autism spectrum disorder among other findings [5]. Recently, Carmignac et al. reported on 19 females carrying 10 novel heterozygous variants, and a wider phenotypic spectrum of the female phenotype emerged [6]. The authors found that all affected individuals presented with learning disabilities or ID, with four also having language impairment. Four of the 19 female carriers were asymptomatic. Interestingly, the authors reported on 5 female patients referred for ID who were found to have a de novo protein-truncating-variants or missense variants. This is largest cohort of female patients reported with de novo variants in this gene.

We report a novel de novo *KDM5C* missense variant (c.1516A > G, p.Met506Val) in a 3-year-old female with global developmental delay, hypotonia and ataxia.

## Case presentation

A 3-year-old female with ataxia, hypotonia and global developmental delay was referred for a neurological evaluation. The proband was conceived naturally, and pregnancy was uncomplicated. She was born full term at 39 weeks gestation via emergent C-section for fetal distress with normal birth parameters [weight 6 lbs. 9 oz. (25th percentile), length 20 in (75th percentile) and head circumference 33.5 cm (25th percentile)]. There were no medical issues or concerns in the newborn period other than a tongue and lip tie which caused feeding issues and was subsequently corrected.

The proband sat at 7 months and walked at 15 months but had significant expressive language impairment; her first word was at 23 months old of age and her speech pattern was notable for scanning speech prosody. During the neurological evaluation at 3 years old, cerebellar findings included dysmetria and wide based jerky gait and she was diagnosed with ataxia. She also was noted to have some anxiety as well by parental report. See Table 1 for EEG, genetic tests and neuropsychological results. MRI was not completed and family history was non-contributory.

**Table 1:** EEG, Genetic Testing and Neuropsychological results

Evaluations	Age at test date	Result
Ambulatory EEG	3 years	Normal
Chromosome Analysis	3 years	Normal (46XX)
Microarray	3 years	Normal
Fragile X	3 years	Normal (29 and 32 CGG repeats)
MECP2 seq + del/dup	3 years	Normal
Trio WES	3 years	De novo missense variant in the *KDM5C* gene (g.X-53239925 T > C; c.1516A > G; p.Met506Val; NM_004187.4)
Wechsler Preschool and Primary Scale of Intelligence - IV	2 years 8 months	Full Scale Intelligence Quotient (IQ) = 85 (Low Average range) General Ability Index = 91 (Average range) Vocabulary Acquisition Index = 75 (Borderline range)^a^
Vineland 3	2 years 8 months	Adaptive Behavior Composite =70 (percentile rank = 2) Communication Domain =72 (percentile rank =3) • The communication domain is made up of two subdomains: Expressive and Receptive. The probands score was *adequate* for receptive and *low* for expressive domains. The probands receptive score is significantly higher than the expressive score. Daily Living Skills Domain = 75 (percentile rank = 5)) Socialization Domain = 65 (percentile rank =1) Motor Skills Domain = 55 (percentile rank < 1) • The motor domain is made of two subdomains Gross and Fine Motor. The probands gross motor was moderately low and fine motor score was low. The gross motor was significantly higher than fine motor
Childhood Autism Rating Scales 2	2 years 8 months	Raw Score = 28.5 (Minimal to no symptoms of Autism Spectrum Disorder)

^a^ Misleading as it represents only rote receptive skills and not communicative ability

The proband and her parents were referred to and enrolled into an IRB approved research whole exome sequencing protocol after signing informed consent (CUIMC IRB # AAAO6702). DNA was extracted from maternal, paternal, and proband blood samples. Libraries were prepared using Kapa Hyper Prep Kit, exome captured with IDT xGen Exome Research Panel v1 and sequenced on the NovaSeq6000. Our process for the filtering and prioritization of variants has been described previously [7]. A de novo missense variant (X-53239925-T-C [GrCh37); c.1516A > G; p.Met506Val) was identified in the *KDM5C* gene in the proband. The presence/absence of the variant was clinically confirmed via Sanger sequencing in the proband, mother and father in a CLIA-approved laboratory [8].

## Discussion and conclusions

In this study, we implicate a novel de novo variant, p.Met506Val, as disease causing in the *KDM5C* gene. To the best of our knowledge, this is only the third female proband identified to harbor a de novo missense variant. Our findings with the neuropsychological tests extend the knowledge of clinical manifestation associated with this rare disorder, by describing a female proband with a *KDM5C* variant showing low average IQ but significant deficiencies in motor and other adaptive skills. Additionally, this is the first report of a female proband with ataxia.

**Fig. 1:**
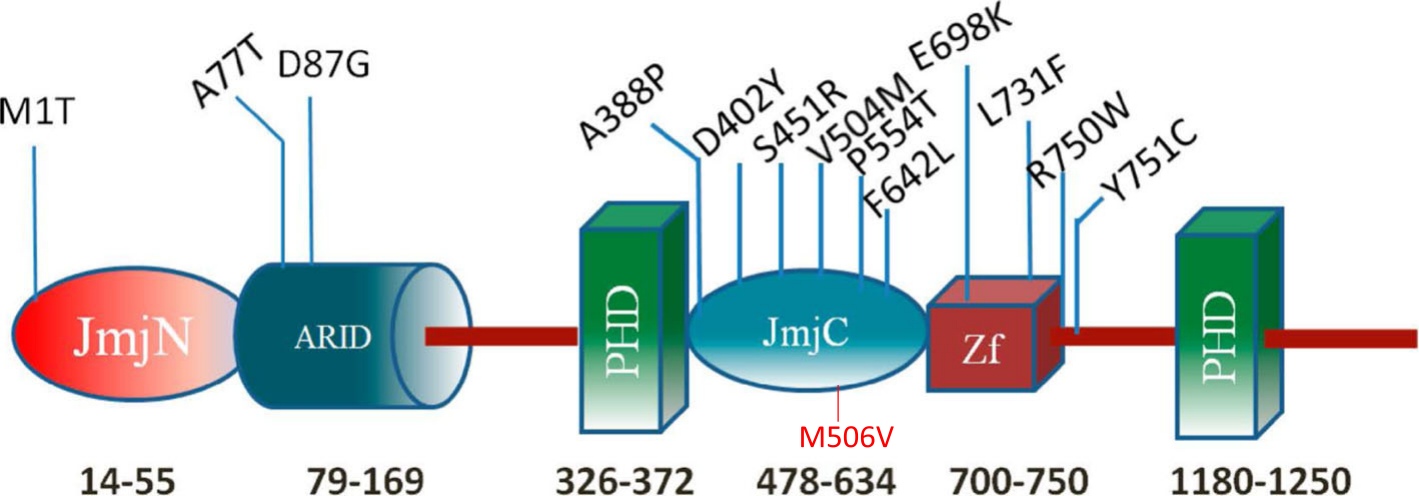
KDM5C protein domains (as shown in PMID# 26580603). The numbers indicate approximate domain boundaries. The known disease-associated missense variants are provided as well. Variant seen in our case is shown in red

The c.1516A > G variant is a single base pair substitution in exon 11 of 26 of the *KDM5C* gene, which causes a substitution of Methionine to Valine at position 506 (506 of 1561) in the jumonji-C domain of this multi domain protein where other missense variants have been previously reported in *KDM5C* related disorders [6]. Jumonji-C domain is the catalytic core for histone H3K4 demethylation and interaction between the JmjC domain is important for the demethylation activity [9, 10]. This c.1516A > G variant is absent from the Genome Aggregation Database (gnomAD) [11] and it meets the hot-zone specific bioinformatics signature described in Zhu et al. [7]. Additionally, this *KDM5C* gene is highly intolerant to functional variation with a pLI score of 1 and a Z-score of 5.15 [11]. Moreover, the CADD (score:25.1;GRCh37-v1.6) and REVEL score (score:0.785; version 4.1) also suggests the variants pathogenicity [12, 13]. Taken together, the evidence supports that the p.Met506Val in *KDM5C* is a strong candidate for disease causation in our proband. This additional variant in *KDMC5* may demonstrate a broader phenotypic spectrum than other genotypes previously reported in the literature (Fig. 1).

There are some notable limitations of this study. First, we cannot exclude a digenic model for this phenotype as other variants have been previously reported with ataxia. We would propose a phenotype spectrum, as opposed to strict genotype-phenotype correlations, especially in the setting of an X-linked disorder. Another limitation is that we have not performed X-inactivation studies to assess whether there was any skewing may have contributed to the phenotype. Further studies to study other epigenic mechanisms may provide a better understanding of the pathophysiology leading to a broader phenotypic spectrum. Importantly, many genetic causes of ataxia are due to expansions such as trinucleotide repeats. While the subject did have FMR repeat testing for Fragile X syndrome, no other repeat expansions were specifically evaluated for as our research study program uses whole exome sequencing strategy for finding causative variants.

Our proband has similar features to other similarly reported female patients with de novo variants in *KDM5C,* presenting with motor delay and language impairment, notably on expressive speech. Other affected female have motor impairments, including oro-facial dyspraxia, oculo-manual coordination difficulties, spasticity and hyperreflexia. Interestingly, our proband does not have any other features that have been reported including a distinctive facial features, skeletal abnormalities, short stature and endocrine features. This could be related to age since some of these features may present in later years. It could also indicate the emerging and variable phenotype associated with *KDM5C* variants in females. This concept is supported by a previous report of a de novo missense variant in *KDM5C* identified in a patient with cerebral palsy [4]. Consistent with this theory is the fact that our proband presented with ataxia at the age of 3 years. To our knowledge, there is no other female patient that has been reported to have a diagnosis of ataxia. There is a report of one family where the male patients had ataxia but interestingly, the affected female relatives were not noted to have this symptom [14].

In conclusion, our case report is consistent with previous reports of language delay as common features in females with *KDM5C* disease causing variants. The detailed neuropsychological testing indicate that our probands motor skills are much more severely affected than her IQ. Moreover, this case report expand the clinical landscape by reporting a female proband with ataxia who harbors a novel de novo variant in *KDM5C*. Overall, this case report further confirm that distinctive facial features, skeletal abnormalities, short stature and endocrine features can be absent in *KDM5C* related disorders. This provides evidence for an emerging and phenotypic variability that adds to the literature of the role of *KDM5C* in females with neurodevelopmental disorders.

## Data Availability

Not applicable.

## Abbreviations

gnomAD: Genome Aggregation Database
ID: Intellectual disability
*KDM5C*: Lysine specific demethylase 5C
